# The Classical Pink-Eyed Dilution Mutation Affects Angiogenic Responsiveness

**DOI:** 10.1371/journal.pone.0035237

**Published:** 2012-05-15

**Authors:** Michael S. Rogers, Victor Boyartchuk, Richard M. Rohan, Amy E. Birsner, William F. Dietrich, Robert J. D’Amato

**Affiliations:** 1 Vascular Biology Program, Children’s Hospital Boston, Boston, Massachusetts, United States of America; 2 Department of Surgery, Harvard Medical School, Boston, Massachusetts, United States of America; 3 Department of Ophthalmology, Harvard Medical School, Boston, Massachusetts, United States of America; 4 Program in Gene Function and Expression, UMass Medical School, Worcester, Massachusetts, United States of America; 5 Novartis Institutes for Biomedical Research, Cambridge, Massachusetts, United States of America; Purdue University, United States of America

## Abstract

Angiogenesis is the process by which new blood vessels are formed from existing vessels. Mammalian populations, including humans and mice, harbor genetic variations that alter angiogenesis. Angiogenesis-regulating gene variants can result in increased susceptibility to multiple angiogenesis-dependent diseases in humans. Our efforts to dissect the complexity of the genetic diversity that regulates angiogenesis have used laboratory animals due to the availability of genome sequence for many species and the ability to perform high volume controlled breeding. Using the murine corneal micropocket assay, we have observed more than ten-fold difference in angiogenic responsiveness among various mouse strains. This degree of difference is observed with either bFGF or VEGF induced corneal neovascularization. Ongoing mapping studies have identified multiple loci that affect angiogenic responsiveness in several mouse models. In this study, we used F2 intercrosses between C57BL/6J and the 129 substrains 129P1/ReJ and 129P3/J, as well as the SJL/J strain, where we have identified new QTLs that affect angiogenic responsiveness. In the case of *AngFq5*, on chromosome 7, congenic animals were used to confirm the existence of this locus and subcongenic animals, combined with a haplotype-based mapping approach that identified the pink-eyed dilution mutation as a candidate polymorphism to explain *AngFq5.* The ability of mutations in the pink-eyed dilution gene to affect angiogenic response was demonstrated using the p-J allele at the same locus. Using this allele, we demonstrate that pink-eyed dilution mutations in *Oca2* can affect both bFGF and VEGF-induced corneal angiogenesis.

## Introduction

Angiogenesis is the process by which new vessels are generated from existing vasculature. In adult tissues it is rare; however it can be initiated by a number of normal and pathological processes including wounding, reproduction, cancer, macular degeneration, and cardiovascular disease. Angiogenesis is regulated by a variety of signaling molecules including VEGF, bFGF, and angiopoietins. There are a number of *in vivo* assays that can measure the angiogenic response to a particular stimulus. Among these is the corneal neovascularization assay. This assay takes advantage of the avascular field of the cornea as a platform in which to induce blood vessel growth. Early studies used tumor pieces to induce this growth, while later assays induced growth using slow-release pellets containing angiogenic growth factors.

Among the earliest described angiogenic proteins is bFGF [1]. bFGF induces angiogenesis largely by activating classical tyrosine kinase signaling pathways downstream of its congnate receptors FGFR1-4, with other pathways also activated [2]. Among the downstream mediators of bFGF–induced angiogenesis in the cornea is VEGF [3]. We have observed significant quantitative differences among common inbred mouse strains in their response to both bFGF [4], [5] and VEGF [3], [6]. Our initial efforts at mapping QTLs for these traits included several F2 intercrosses. Here we report the results of F2 mapping studies, including the identification of a new genetic modulator of angiogenic signaling. Efforts involving recombinant-inbred strains that were initiated later, but completed earlier have been reported previously [3], [4].

The pink-eyed dilution mutation in the *Oca2* gene contained in the *P* locus is one of the oldest described in mice and, along with the albino mutation in tyrosinase, was involved in the earliest descriptions of linkage [7]. The mutation arose in the Asiatic mouse substrain *Mus musculus molossinus* and was introduced into common laboratory strains as a result of the 19^th^ century mouse trade [8]. A wide variety of additional alleles are known at the *P* locus, all of which result in some level of hypopigmentation in mice. In humans, polymorphisms in an enhancer upstream of *OCA2* play a major role in determining eye color. Individuals bearing the less active C/C genotype at rs12913832 exhibit lighter/blue eye color [9]. In addition, an R419Q polymorphism in the protein itself also results in lighter eye color [9].

Pigmentation is the result of the production of melanin in cells. The rate limiting step in this process is the oxidation of tyrosine to dopaquinone, which is catalyzed by tyrosinase (the product of the c locus). Dopaquinone is then rearranged and further oxidized by sequential actions of dopachrome tautomerase and tyrosinase-related protein 1 (the product of the b locus) [10]. The resulting molecules spontaneously polymerize into the black pigment eumelanin. Alternatively, in the absence of the latter two enzymes, the reaction of dopaquinone with cysteine or glutathione leads to the production of the sulfur-containing, reddish-brown pigment pheomelanin [10]. The role of the Oca2 protein in this process is currently incompletely defined. It is homologous to several 12-transmembrane-domain transporter proteins [11]. In melanocytes lacking active p-protein, tyrosinase fails to mature and localize properly [12]. This results in protein cleavage and secretion of active tyrosinase and melanin. As a result, mice bearing the p-mutation show higher circulating levels of pigment precursors such as 5-*S*-cysteinyldopa (5-*S*-CD) and dihydroxyindolecarboxylic (DHICA) acid than fully pigmented animals [13].

Here we outline studies aimed at identifying the nature of inheritance of angiogenic responsiveness, identifying loci that control for that responsiveness, and identifying specific genetic changes within one of those loci that affect the angiogenic response.

## Results

**Figure 1:**
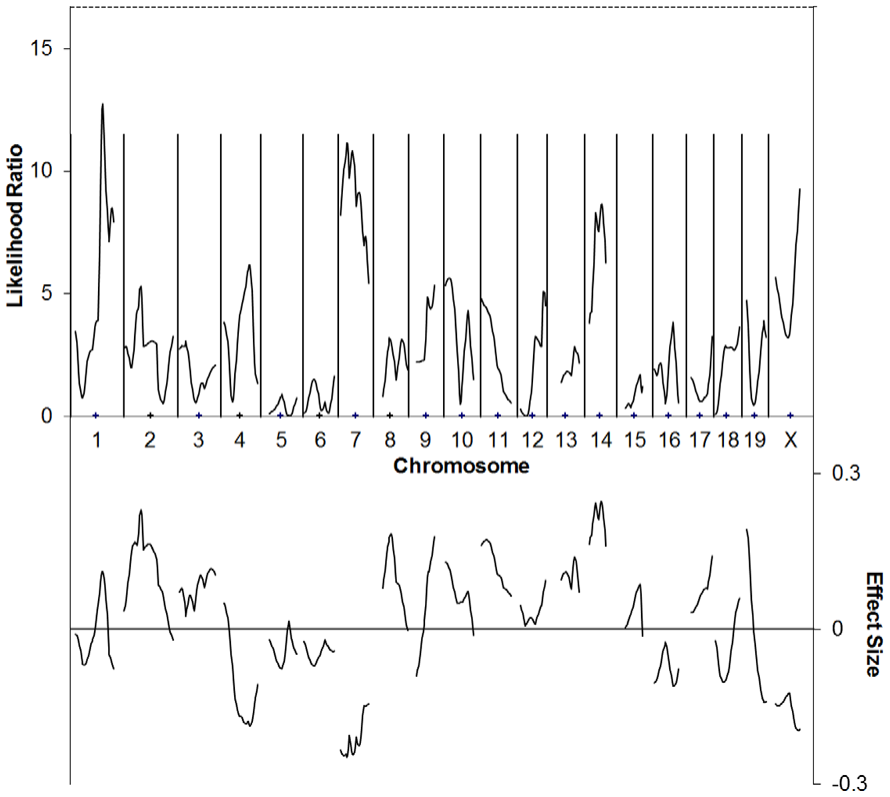
C57BL/6J×129P1/ReJ F2 cross simple interval mapping. Whole genome simple interval map. Top: likelihood ratio statistic or likelihood of a region being linked to bFGF-induced corneal neovascularization. Dashed line indicates P = 0.05 as determined by bootstrapping. Bottom: predicted additive effect of a region on bFGF-induced corneal neovascularization.

In all our crosses we used C57BL/6J as one of the parental strains. Our initial attempt at mapping differences in angiogenic responsiveness was done using 129P1/ReJ mice. The angiogenic response of F2 animals was determined using the corneal neovascularization assay [14] using a dose of 10 ng bFGF. This dose was chosen because it would keep the angiogenic response of both parental strains within the linear range of the assay (∼0.4–2.2 mm2) [14] and thus was likely to keep most of the progeny within this range as well. We had hoped that the large phenotypic difference between this strain and C57BL/6J was the result of a few polymorphisms in major genes that would be easily identified, however this was not the case. In subsequent years, we have identified a large number of loci that affect angiogenesis [3], [4], [15], [16] which may explain why no individual strong loci were identified in the C57BL/6J×129P1/ReJ cross. Nevertheless, by analyzing 77 F2 animals from this cross, we observed markers with uncorrected p values <0.05 for association between vessel area and genotype on chromosomes 1 (near *D1Mit218*), 7 (between *D7Mit76* and *D7Mit105*), 10 (near *D10Mit298*), 11 (near *D11Mit78*), 14 (between *D14Mit234* and *D14Mit229*, and X (See Figure 1/Table 1). To assess whether any of these loci exhibited genome-wide statistical significance, we turned to interval mapping. This demonstrated that regions on Chromosomes 1 and 7 exhibited LR scores greater than the traditional cut-off for suggestive linkage of 11.5 (equivalent to a LOD score of 2.5), however permutation testing revealed that none of these was significant at the genome-wide level. Composite interval mapping showed an additional region of linkage on the proximal portion of chromosome 2 that may correspond to *AngVq2* [4]. (Figure S1).

**Figure 2:**
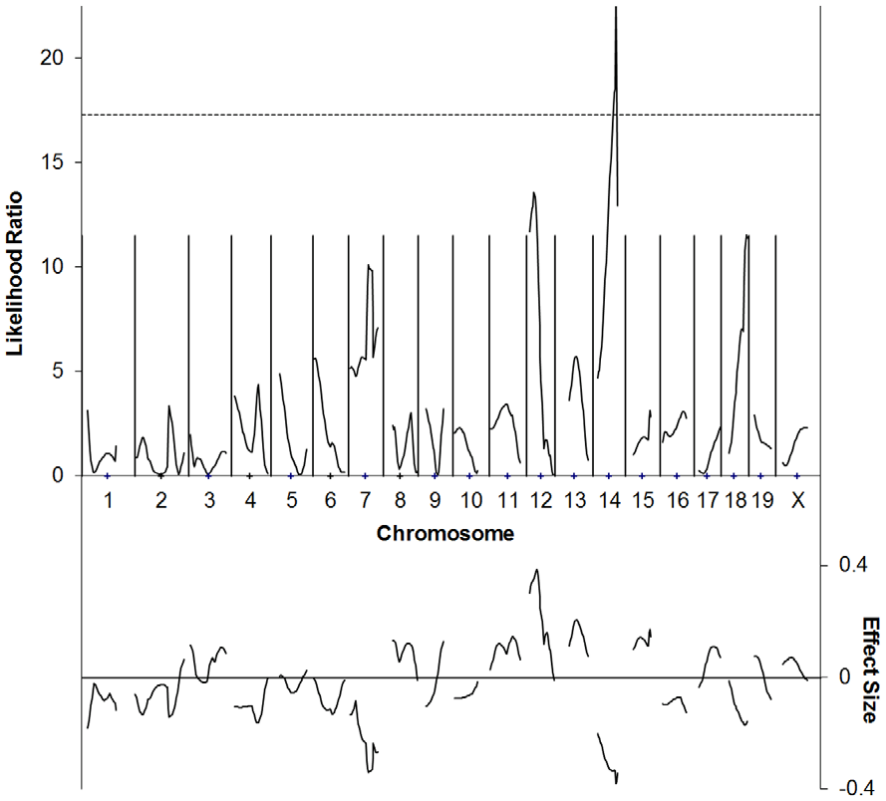
C57BL/6J×129P3/J F2 cross simple interval mapping. Whole genome simple interval map. Top: likelihood ratio statistic or likelihood of a region being linked to bFGF-induced corneal neovascularization. Dashed line indicates P = 0.05 as determined by bootstrapping. Bottom: predicted additive effect of a region on bFGF-induced corneal neovascularization.

The next cross examined was with 129P3/J, which was also assessed at 10 ng bFGF. Simple association tests for each of the markers demonstrated uncorrected p values <0.05 for regions on chromosome 7, proximal 12 (up to *D12Mit46*), and 14 (between *D14Mit228* and *D14Mmit198*). (See Figure 2/Table 2). In interval mapping, the linked region on chromosome 12 exhibited the strongest effect, with an estimated effect on vessel area of ∼0.6 mm^2^. On Chr. 14, we found linkage to a region centered on *D14Mit265* that appears to reduce vessel area by about 0.4 mm^2^ in 129 mice. This region exceeded the genome-wide permutation threshold for p<0.05 of 17.3. Weak linkages in this cross that are of interest because they may confirm linkage identified in other crosses, include one at *D18Mit188*. This region corresponds to *AngFq4* in the BXD RI cross [3]. We also see a weak linkage peak on Chromosome 7 in this cross.

**Table 1:** Markers showing near-significant and significant association with bFGF-induced corneal neovascularization in a 77-animal C57BL/6J×129P1/ReJ F2 cross.

Marker	Chrom.	cM	b0	b1	F	%	P	
D1Mit218	1	67	0.708	0.19	8.979	11%	0.004	**
D2Mit396	2	69	0.712	0.119	2.805	4%	0.098	
D7Mit76	7	3.4	0.713	−0.173	6.807	8%	0.011	*
D7Mit246	7	15	0.707	−0.193	10.01	12%	0.002	**
D7Mit229	7	23	0.706	−0.189	10.05	12%	0.002	**
D7Mit201	7	37	0.702	−0.184	8.595	10%	0.004	**
D7Mit253	7	53	0.709	−0.181	6.933	8%	0.01	*
D7Mit105	7	64	0.715	−0.162	5.062	6%	0.027	*
D10Mit298	10	3	0.715	0.151	5.028	6%	0.028	*
D11Mit78	11	2	0.727	0.128	4.167	5%	0.045	*
D12Mit150	12	59	0.707	0.123	3.775	5%	0.056	
D14Mit152	14	15	0.708	0.129	3.006	4%	0.087	
D14Mit234	14	23	0.692	0.203	7.634	9%	0.007	**
D14Mit30	14	29	0.699	0.191	7.504	9%	0.008	**
D14Mit39	14	30	0.699	0.191	7.504	9%	0.008	**
D14Mit228	14	46	0.697	0.151	5.072	6%	0.027	*
D16Mit139	16	43	0.706	−0.14	3.512	4%	0.065	
D17Mit155	17	56	0.719	0.118	3.193	4%	0.078	
D19Mit128	19	11	0.715	0.144	3.88	5%	0.053	
DXMit166	20	16	0.708	−0.11	3.984	5%	0.05	*
DXMit186	20	69	0.702	−0.139	6.54	8%	0.013	*

Marker = marker tested; Chr. = chromosome the marker is on; % = fraction of the experimental variance attributable to genotype at the marker indicated under an additive model; P = likelihood that there is no relationship between the marker genotype and choroidal neovascularization area (by F test), when the data are fit to the simple linear regression model y = b0+b1×+e. The results give the estimates for b0, b1 and the F statistic for each marker. b0 is approximately the average area of C57BL/6J-allele-containing strains. b1 is an indication of the effect of substitution of the 129P1/ReJ allele at that marker. Asterisks indicate uncorrected P<0.05 (*), P<0.01 (**), or P<0.001 (***).

**Figure 3:**
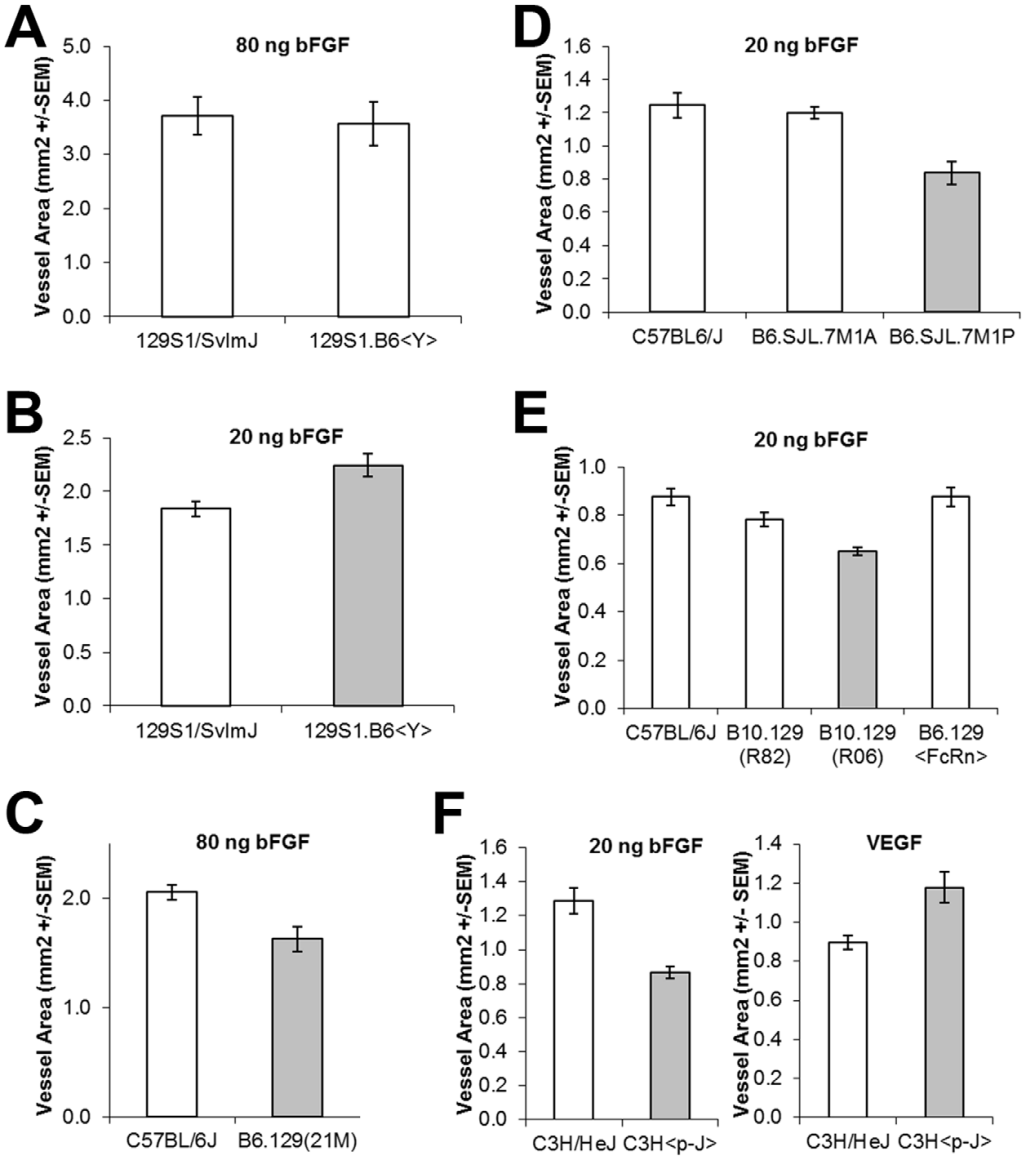
Angiogenic response of Congenic Strains. Differences between parental (leftmost) and congenic strains are statistically significant (P<0.05) except when the bar for the congenic strain is colored white.

To test the role of parental sex we did a reciprocal cross using C57BL/6J and 129P3/J animals and found that, in males only, the F1 value was dependent on the genetic background of the father, suggesting that in this cross one of the sex chromosomes bears a locus that affects angiogenesis (Figure S2). This was confirmed using the 129S1.B6<Y> strain (a kind gift of Dr. Joseph Nadeau) which has the C57BL/6J Y chromosome on a 129S1/SvImJ background. When angiogenesis in these strains was compared using experiments with 80 ng bFGF pellets, we observed no significant difference in angiogenic response, likely because the response of both strains was outside the linear range of the assay. Consistent with this notion, when the angiogenic response of 129S1.B6<Y> was compared with that of the 129S1/SvImJ parent using a 20 ng bFGF pellet, we observed a 22% increase in angiogenic response, consistent with the difference observed between F2 animals with 129 vs. B6 Y chromosomes (Figure 3a,b).

**Table 2:** Markers showing near-significant and significant association with bFGF-induced corneal neovascularization in a C57BL/6J×129P3/J F2 cross.

Marker	Chrom.	cM	b0	b1	F	%	P	
D7Mit76	7	3.4	0.76	−0.163	4.943	7%	0.03	*
D7Mit246	7	15	0.757	−0.142	3.636	5%	0.061	
D7Mit318	7	37	0.755	−0.168	4.491	7%	0.038	*
D7Mit62	7	43	0.751	−0.213	7.008	10%	0.01	*
D7Mit126	7	50	0.76	−0.189	5.470	8%	0.023	*
D7Mit105	7	64	0.76	−0.168	4.482	7%	0.038	*
D12Mit105	12	6	0.783	0.258	11.97	16%	0.001	***
D12Mit136	12	13	0.788	0.268	12.33	16%	0.001	***
D12Mit46	12	16	0.794	0.254	11.88	16%	0.001	**
D12Mit34	12	29	0.793	0.143	3.309	5%	0.074	
D14Mit14	14	10	0.784	−0.144	3.290	5%	0.074	
D14Mit182	14	14	0.783	−0.154	3.653	5%	0.061	
D14Mit228	14	46	0.766	−0.165	5.315	8%	0.024	*
D14Mit265	14	48	0.763	−0.184	6.486	9%	0.013	*
D14Mit198	14	54	0.766	−0.177	5.265	8%	0.025	*
D14Mit75	14	54	0.758	−0.142	3.482	5%	0.067	

Marker = marker tested; Chr. = chromosome the marker is on; % = fraction of the experimental variance attributable to genotype at the marker indicated under an additive model; P = likelihood that there is no relationship between the marker genotype and choroidal neovascularization area (by F test), when the data are fit to the simple linear regression model y = b0+b1×+e. The results give the estimates for b0, b1 and the F statistic for each marker. b0 is approximately the average area of C57BL/6J-allele-containing strains. b1 is an indication of the effect of substitution of the 129P3/J allele at that marker. Asterisks indicate uncorrected P<0.05 (*), P<0.01 (**), or P<0.001 (***).

Our final crosses involved SJL/J and C57BL/6J mice. We performed three distinct F2 crosses (72, 66, and 186 animals) between these strains resulting in 325 animals available for analysis. The angiogenic response of the resulting animals was determined using the corneal neovascularization assay [14] using a dose of 80 ng bFGF. As with the other crosses, the data, as measured by vessel area, showed a distribution that did not differ significantly from normality as determined by a Shapiro-Wilk test.

**Figure 4:**
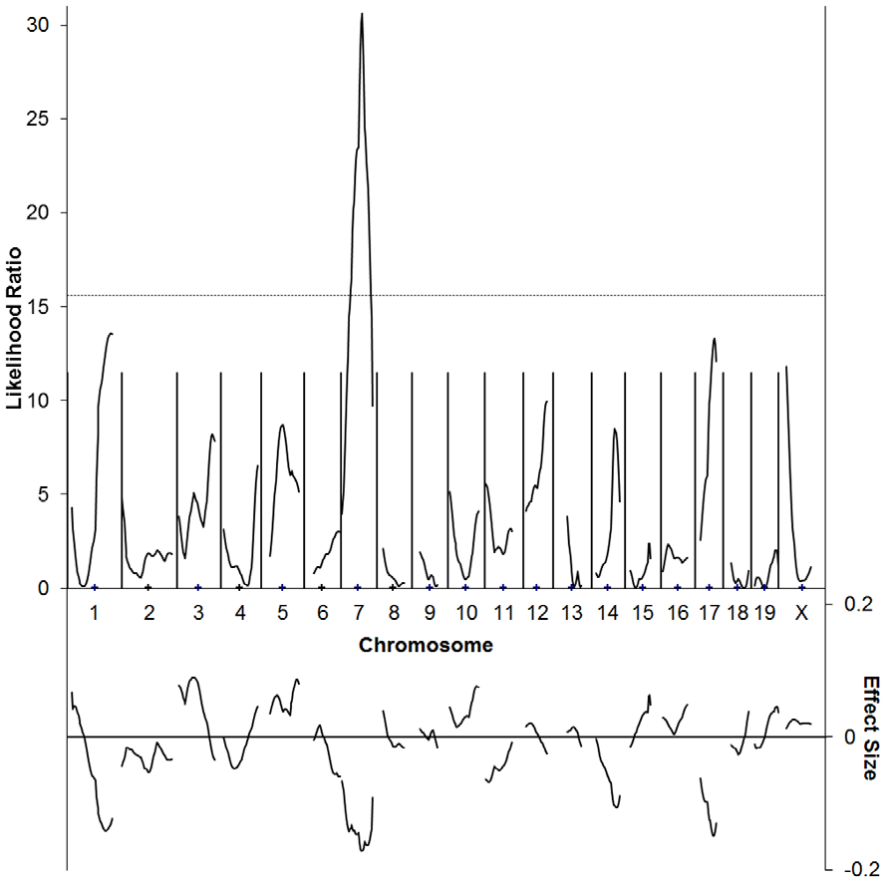
C57BL/6J×SJL/J F2 cross simple interval mapping. Whole genome simple interval map. Top: likelihood ratio statistic or likelihood of a region being linked to bFGF-induced corneal neovascularization. Dashed line indicates P = 0.05 as determined by bootstrapping. Bottom: predicted additive effect of a region on bFGF-induced corneal neovascularization.

The genotype of these animals at 112 loci across the genome was determined using microsatellite markers. In the initial cross (73 animals), association was observed between 15 markers and differences in angiogenic response (Table S1). Interval mapping using data from the first cross demonstrates statistically significant linkage to distal chromosome 5 (between *D5Mit158* and *D5Mit99*) and proximal chromosome 7 (near *D7Mit20*, Figure S3). Two additional crosses were undertaken to confirm these results. As animals from these crosses were added to the map, linkage to chromosome 7 was strengthened, while linkage to chromosome 5 was weakened until it was no longer significant. In the complete data set, association with an uncorrected P<0.05 between animal genotype and vessel area was observed at markers on chromosomes 1, 3, 5, 7, 11, and 17 (Table 3). Interval mapping on the full data set revealed a single region of genome-wide highly significant linkage on chromosome 7 that can explain approximately 10% of the variance in this cross (Figure 4). This peak was present when using several different measures of corneal angiogenesis, indicating that the mapping is not sensitive to the precise measure of vessel growth used (Figure S4). The use of more advanced mapping techniques, including composite interval mapping [17], [18], and multiple interval mapping [19] failed to reveal additional areas of statistically significant linkage, though several regions of suggestive linkage were identified.

**Table 3:** Markers showing near-significant and significant association with bFGF-induced corneal neovascularization in a C57BL/6J×SJL/J F2 cross.

Marker	Chrom.	cM	b0	b1	F	%	P	
D10Mit44	1	9	1.404	0.066	4.314	1%	0.039	*
D1Mit3	1	11	1.403	0.053	2.955	1%	0.087	
D1Mit306	1	59	1.401	−0.051	2.851	1%	0.092	
D1Mit94	1	64	1.4	−0.069	5.176	2%	0.024	*
D1Mit36	1	92	1.395	−0.107	13.23	4%	0.000	***
D3Mit51	3	35	1.41	0.065	4.147	1%	0.043	*
D3Mit106	3	55	1.405	0.052	2.862	1%	0.092	
D5Mit205	5	45	1.409	0.077	6.144	2%	0.014	*
D5Mit338	5	59	1.407	0.061	4.236	1%	0.040	*
D5Mit158	5	62	1.407	0.073	5.336	2%	0.022	*
D5Mit188	5	64	1.407	0.077	5.645	2%	0.018	*
D5Mit168	5	78	1.403	0.061	4.261	1%	0.040	*
D5Mit99	5	80	1.403	0.06	3.776	1%	0.053	
D6Mit59	6	67	1.401	−0.057	3.045	1%	0.082	
D6Mit294	6	73	1.401	−0.051	2.845	1%	0.093	
D7Mit76	7	3.4	1.402	−0.061	3.919	1%	0.049	*
D7Mit246	7	15	1.401	−0.111	10.99	3%	0.001	**
D7Mit270	7	18	1.4	−0.123	14.77	4%	<10^−3^	***
D7Mit229	7	23	1.4	−0.121	16.77	5%	<10^−4^	****
D7Mit145	7	26	1.4	−0.132	20.46	6%	<10^−5^	****
Oca2	7	28	1.4	−0.135	20.98	6%	<10^−5^	****
D7Mit318	7	37	1.404	−0.141	24.18	7%	<10^−5^	****
Tyr	7	44	1.407	−0.163	31.23	9%	<10^−7^	****
D7Mit126	7	50	1.405	−0.155	25.3	7%	<10^−6^	****
D7Mit105	7	64	1.401	−0.11	13.03	4%	<10^−3^	***
D7Mit12	7	66	1.4	−0.089	8.23	2%	0.004	**
D10Mit180	10	64	1.405	0.061	3.822	1%	0.051	
D11Mit78	11	2	1.402	−0.073	5.454	2%	0.020	*
D17Mit66	17	25	1.402	−0.071	4.847	1%	0.028	*
D17Mit88	17	30	1.402	−0.09	8.212	2%	0.004	**
D17Mit93	17	45	1.401	−0.066	4.624	1%	0.032	*
DXMit166	20	16	1.412	0.055	3.676	1%	0.056	

Marker = marker tested; Chr. = chromosome the marker is on; % = fraction of the experimental variance attributable to genotype at the marker indicated under an additive model; P = likelihood that there is no relationship between the marker genotype and choroidal neovascularization area (by F test), when the data are fit to the simple linear regression model y = b0+b1×+e. The results give the estimates for b0, b1 and the F statistic for each marker. b0 is approximately the average area of C57BL/6J-allele-containing strains. b1 is an indication of the effect of substitution of the 129P3/J allele at that marker. Asterisks indicate uncorrected P<0.05 (*), P<0.01 (**), P<0.001 (***) or P<0.0001 (****).

Based on the results from the first cross, animals congenic for the linked region on chromosome 5 and similar congenics for the chromosome 7 region were generated by repeated backcross of males heterozygous for these regions to C57BL/6J females. Because of decreasing support for linkage on chromosome 5 in the second and third F2 crosses, backcrossing for these congenics was terminated at seven generations. These animals showed no difference in angiogenic response when compared to C57BL/6J controls using bFGF-induced corneal neovascularization as the measure (Figure S5). We thus conclude that there are no genetic polymorphisms between SJL/J and C57BL/6J animals that alter bFGF-induced angiogenesis within this region of chromosome 5.

**Figure 5:**
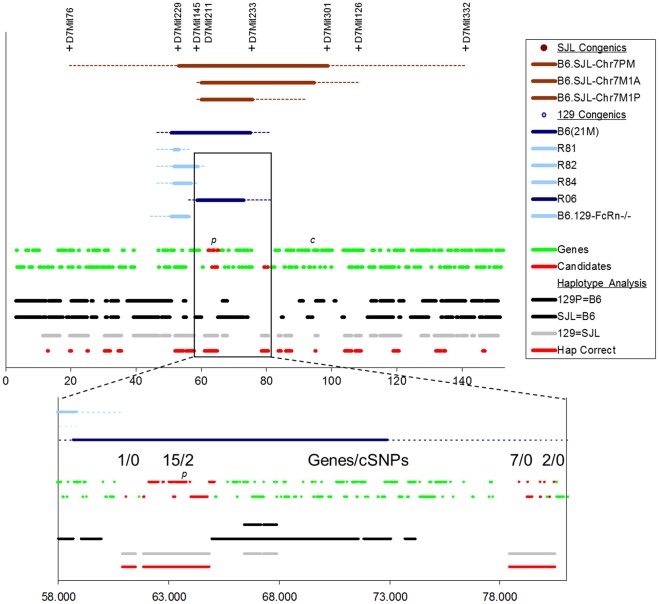
Mapping the region responsible for *AngFq5*. Top: Location of the congenic regions for the congenic strains used is plotted, along with boundary-defining markers for SJL congenics (brown). Blue indicates 129 congenics, with dark blue indicating those congenics that demonstrate a statistically significant difference in angiogenic response. Genes are indicated next by which DNA strand they are coded by. Finally regions of shared haplotype are indicated colored by whether the parental strains have disparate (black) or shared (grey) alleles at *AngFq5*. Below: Magnification of the region of interest defined by the R82 and R06 congenics. The number of genes, and genes bearing amino-acid altering polymorphisms (cSNPs), is indicated as are the locations of the pink-eyed dilution gene (*p*) and the tyrosinase gene (*c*). Scale is in Mbp on build 37.1 of mouse Chromosome 7.

Several chromosome 7 congenics (Figure 5) were also generated as outlined in Materials and Methods. These strains exhibited a decrease in bFGF-induced angiogenesis when compared to C57BL/6J controls, with the B6 allele exhibiting partial dominance (Figure S6). In addition, intercrosses of 8^th^ generation backcross animals which contained SJL/J congenic regions on both chromosome 7 and the Y chromosome were further suppressed, when compared to animals containing either region alone, indicating that the chromosome 7 region and the Y chromosome region exhibit additive effects. Together, these results indicate that there are genetic differences between SJL/J and C57BL/6J animals on chromosome 7 near the pink-eyed dilution locus that affect the angiogenic response. We have named the chromosome 7 QTL *AngFq5*.

Classical mouse strains including the C57, SJL, and 129 strains are the result of inbreeding of a few small populations. As a result they share large genomic regions that are identical by descent. Using data from the mouse HapMap project, we determined that a genomic region surrounding the pink-eyed dilution locus is shared by several strains including SJL/J, 129P2/OlaHsD, and 129X1/SvJ. In this analysis, data from the closely related 129P2/OlaHsD strain was used in place of 129P3/J and 129P1/ReJ strain data because dense genotyping was unavailable at the time for the latter strains.

Based on the observation of shared haplotype among these strains, and the observation that both of the 129 substrain crosses identified linkage to the same region on chromosome 7 (albeit at local, but not genome-wide, significance levels), we sought to determine whether C57BL mice bearing 129 congenic regions might also bear the SJL allele of *AngFq5*. We obtained the B10.129P(21 M) congenic line [20], as well as several subcongenic lines (designated R81, R82, R84, and R06) from Dr. D. Roopenian (Jackson Laboratories, Bar Harbor Maine). In addition, we obtained the B6.129X1-*Fcgrt^tm1Dcr^* strain (labeled B6.129<FcRn> in the figure) which contains a knocked out Fc-gamma receptor, as well as a portion of the surrounding 129X1/SvJ genome on a C57BL/6J background. Corneal neovascularization assays on these strains demonstrated that B10.129P(21 M) and R06 show reduced angiogenic responsiveness compared to control animals and thus that they bear the SJL allele at *AngFq5* (Figure 3C,E). Analysis of the congenic regions in these strains reduced the *AngFq5* critical region to approximately 22 Mbp (Figure 5). Of this, ∼75% was deprioritized either because SJL/J shares haplotype with C57BL/6J, or because SJL/J does not share haplotype with 129P2/OlaHsD (a close relative of the 129P3/J and 129P1/ReJ strains used in the initial crosses). This resulted in 4 regions of appropriate haplotype containing 1, 15, 7, and 2 annotated genes distributed across a total of 5.5 Mbp. Of these, only 2 exhibited SNPs predicted to alter amino-acid sequence (cSNPS). *Herc2* (hect domain and RCC1-like domain 2) exhibited a K3566R polymorphism and *Oca2* exhibited the L266V polymorphisms that results in the pink-eyed dilution phenotype (Figure 5).

To directly test whether a pink eyed dilution mutation can suppress angiogenesis, we used the p-J mutation. This mutation arose spontaneously at The Jackson Laboratory on a C3H background. As a result, C3H and C3H^p–J^ represent a pair of strains that differ only in a deletion at the pink-eyed dilution locus (i.e. there are no linked loci to confound analysis). A comparison of the bFGF-induced vessel area in C3H and C3H^p–J^ animals shows that a pink-eyed dilution mutation suppresses angiogenesis to an extent that is sufficient to explain *AngFq5* (Figure 3F). Interestingly, in contrast to bFGF, VEGF-induced angiogenesis is potentiated by this mutation, suggesting differences in the mechanism by which these two angiogenesis signaling pathways are affected by the pink-eyed dilution mutation. This is noteworthy because previous results from BXD strains in our lab showed a strong correlation between bFGF and VEGF-responsiveness [3], indicating that most angiogenesis-controlling QTLs in that cross affect bFGF and VEGF similarly. Importantly both parents in that cross bear the wild-type pink-eyed dilution allele. Thus, the trait doesn’t segregate in that cross.

It is also noteworthy that in contrast to intercross F2 animals (which include animals heterozygous at one or both of the *Tyr* and *Oca2*. loci), fully homozygous B6.SJL.M1A animals showed no decrease in angiogenic response relative to C57BL/6J controls, while B6.SJL.M1P animals, which contain a smaller region of SJL chromosome 7 did show a decrease in angiogenic responsiveness (Figure 3D). This suggests that the portion of SJL chromosome 7 not included in B6.SJL.M1P animals had either a compensating or epistatic interaction with the polymorphism responsible for *AngFq5*. Importantly, in the coat color phenotype the albino mutation at tyrosinase interacts epistatically with pink-eyed dilution mutation in *Oca2*. Animals bearing the albino mutation are albino in appearance regardless of their genotype at *Oca2*. We have observed that, animals bearing an albino mutation show no difference in corneal angiogenic response to bFGF [5]. Thus, the absence of a difference in angiogenic response in B6.SJL.M1A animals indicates that similar epistatic interactions occur in the case of angiogenic responsiveness. It further suggests that soluble biochemical products of pigment production play a role in the differences in angiogenic response observed.

## Discussion

Both previously published data, and the data presented here clearly demonstrate that angiogenic responsiveness is controlled by a large number of different polymorphic genes. It may be instructive that *Oca2* is not part of a classical angiogenesis-regulatory pathway. This may indicate that a wide variety of angiogenesis-regulatory pathways remain to be discovered. In addition, it may be the consequence of the importance of angiogenesis in reproduction and development. As a result, polymorphisms in pathways that are central to angiogenesis may be incompatible with long-term strain viability. Indeed, VEGF gene knockout mice exhibit haploinsufficiency [21] and low expressing polymorphisms in the VEGF gene are associated with reproductive difficulties (reviewed in [16]).

Repeated identification of similar regions in independent crosses is a useful means of establishing the robustness of linkage between genomic regions and phenotypic traits. When the crosses are between different strains, it has the added advantage of identifying alleles that are shared between the differing strains at a particular locus. This information can be used to identify appropriate haplotypes bearing the alleles in question. We observed overlap between linked regions in these crosses and two previously identified QTLs. In the C57BL/6J×129P3/J cross, we see linkage indicating that 129P3/J bears the DBA/2J allele at *AngFq4* [3]. Similarly we show evidence that 129P1/ReJ bears the DBA/2J allele at *AngVq2* [4]. As outlined in the results section, we also see overlap in linked regions in several of the crosses described here. 129P1/ReJ and 129P3/J share alleles at a locus on chromosome 14 that we name *AngFq7*. Similarly, an angiogenesis-regulating polymorphism on the Y chromosome in several 129 strains results in *AngFq8*. As outlined above, haplotype analysis allowed the identification of the SJL allele of the *Oca2* gene as a strong candidate for *AngFq5*.

The mechanism by which the *Oca2* gene might affect angiogenesis is currently unclear, especially since the cornea does not contain melanocytes. It implies an effect that extends beyond the local microenvironment such that iris melanocytes or skin melanocytes release factors which affect the corneal limbal vessels. For example, circulating melanin precursors might mediate the effect on corneal angiogenesis. Alternatively, because improperly trafficked tyrosinase is secreted by melanocytes bearing the *Oca2^p^* allele [12] and tyrosinase readily generates reactive oxygen [22], increased circulating tyrosinase may lead to increases in reactive oxygen species. This reactive oxygen can, in turn, stimulate endothelial cell production of VEGF as well as other endothelial cell activity associated with angiogenesis [23], [24]. Other endothelial cell signaling molecules affected by ROS include phosphatases, transcription factors and bioactive lipids [25], [26].

In addition, circulating tyrosinase has the capacity to alter circulating levels of catecholamines. Spontaneous disproportination of dopaquinone yields DOPA as one of its products. In addition, *met*-tyrosinase consumes DOPA or dopamine as it is returned to an active redox state. Finally, in addition to tyrosine, tyrosinase can use a variety of phenols and catechols as substrates. Since different catecholamines can produce both pro- and antiangiogenic effects [27], [28], [29], circulating tyrosinase has the potential to significantly alter the angiogenic response. Similar differences in these known angiogenesis modulators may explain the differential response to bFGF, due to differential effects on the timing and nature of signaling. Alternatively these differences might be explained by regulation of novel angiogenesis signaling molecules by the pink-eyed dilution protein.

In humans polymorphisms in the human homolog of the *Oca2* gene are associated with genetic susceptibility to melanoma [9]. Importantly, this effect may not be entirely dependent on differences in pigmentation. *OCA2* polymorphisms are associated with differences in eye color, however, *OCA2*-linked differences in melanoma susceptibility persist after controlling for eye color [30]. This indicates that differences in pigmentation and therefore UV susceptibility may not be sufficient to explain the difference in melanoma susceptibility. It has been suggested that subtle differences in the quality of melanin produced, or differences in glutathione metabolism observed in *Oca2*-mutant melanocytes may account for differing disease susceptibility [30]. To this, we add the possibility that increased VEGF angiogenic responsiveness may affect genetic susceptibility to melanoma. An increased angiogenic response in individuals with appropriate *OCA2* genotype may allow earlier conversion of melanoma to three-dimensional growth and thus frank disease. Extrapolating this idea to angiogenic responsiveness in general suggests that an increased identification and understanding of angiogenesis response alleles may allow improved identification of individuals susceptible to angiogenesis dependent diseases such as cancer.

## Materials and Methods

### Ethics Statement

All animal studies were conducted according to protocols A00-06-046R, A03-10-075R, A06-09-078R, and A09-09-1511R approved by the Institutional Animal Care and Use Committee of Children’s Hospital.

### F2 Crosses

A total of 65 F2 animals were generated by intercrossing F1 progeny of 129P1/ReJ and C57BL/6J animals obtained from Jackson Labs. The resulting animals were genotyped using 89 publicly available SSLP markers. These animals were then phenotyped using the corneal neovascularization assay [14]. For the 129P3/J F2 cross, we used B6129PF1/J animals commercially available from Jackson Labs as parents. A total of 77 F2 animals were generated, genotyped at 98 SSLP markers, and phenotyped as above. In both 129 crosses, animals with a high inter-eye variability (coefficient of variation >0.45) were excluded from further analysis. C57BL/6J x SJL/J F2 animals were generated from grandparents obtained from Jackson labs. These animals were genotyped at 112 publicly available SSLP markers and phenotyped as above.

### Generation of Congenic Animals

B6.SJL chromosome 5 congenics were generated from C57BL/6J x SJL/J F1 animals by selecting males in each generation that were heterozygous at *D5Mit158*, *D5Mit188*, *D5Mit213*, *D5Mit168*, and *D5Mit43*. These animals were backcrossed to C57BL/6J females for seven generations. After the seventh backcross generation, the animals were intercrossed to generate homozygous congenics.

Chromosome 7 congenics were generated in a similar manner except that animals heterozygous for *D7Mit229*, *D7Mit211*, *D7Mit233*, *D7Mit62*, and *D7Mit301*, but homozygous for the B6 allele at *D7Mit332* were selected, and backcrossing was continued for a full ten generations before intercrossing. In addition, at the 8^th^ and 9^th^ backcross generations, three additional lines were generated. B6.SJL^ChrY^ animals were generated by selecting an N8 animal that was homozygous for the B6 allele at all of the chromosome 7 markers for further backcrossing. B6.SJL.7M1A animals were generated from an N8 animal that had a recombination event between *D7Mit229* and *D7Mit 233*. This resulted in animals that were B6 for the proximal portion of Chromosome 7 and had an albino coat color. B6.SJL.7M1P animals were generated from a B6.SJL.7M1A heterozygous animal that experienced a recombination between *D7Mit233* and *D7Mit62*, resulting in an animals that retained the SJL/J pink-eyed dilution allele, but had the C57BL/6J tyrosinase allele, resulting in animals that were pink-eyed dilute in coat color.

### Mapping

Windows QTL Cartographer version 2.5 [31] was used throughout for linkage analysis. Default settings were used throughout. Marker genotypes were obtained using PCR and agarose gel electrophoresis. Primers for SSLP markers and associated linkage map positions were obtained from MGI version 4.21. Simple association, interval mapping [32], and composite interval mapping [17], [18] were all performed. Permutation analysis using 10,000 iterations was performed for each of the three interval mapping experiments, resulting in threshold likelihood ratios (LR) for P<0.05 of 16.7, 17.3, and 15.6 for the C57BL/6J cross with 129P3/J, 129P1/ReJ, and SJL/J respectively. In the case of the SJL cross, P<0.001 corresponded to a LR of 24.8.

### Haplotype Mapping

For haplotype mapping genotypes for C57BL/6J, C57BL/10J, 129P2/OlaHsD, 129X1/SvJ, and SJL/J strains were obtained from the Broad institute hapmap website on 22 July 2009 (http://www.broadinstitute.org/mouse/hapmap/). This file contains SNP position information relative to NCBI build 37 of the mouse genome, as well as genotype information for 94 different strains. Regions of shared haplotype were determined by identifying regions where flanking polymorphic SNPs were on average >1 MB from a given SNP [15]. Uncalled SNPs were not included in this analysis.

### Corneal Micropocket Assay

The corneal micropocket assay was performed as described [14], [33] using pellets containing the indicated quantity of FGF2 or 160 ng carrier free human recombinant VEGF 165 (R&D Systems, Minneapolis, Minnesota). The area of vascular response was assessed on the fifth (FGF2) or sixth (VEGF) postoperative day using a slit lamp. Vessel area was calculated using the formula 0.2π×VL×CH where VL is vessel length from the limbus in mm and CH is clock hours around the cornea. Control C57BL/6J (typically 5 mice, 10 eyes) were included in each assay to confirm consistency. All mouse strains were obtained from Jackson Laboratories (Bar Harbor, Maine) and were housed in Children’s Hospital’s animal facility on standard diet and bedding until the assay was performed.

## Supporting Information

Figure S1 **Composite interval mapping of the C57BL/6J×129P1/ReJ F2 cross.** Whole genome composite interval map. Top: likelihood ratio statistic or likelihood of a region being linked to bFGF-induced corneal neovascularization. Bottom: predicted additive effect of a region on bFGF-induced corneal neovascularization. The indicated likelihood ratio of 11.5 is equivalent to a LOD score of 2.5.
(TIF)

Figure S2 **Effect of parental gender on vessel area in a 129×B6 F1 cross.** Pellets contain 10 ng bFGF. The increase in vessel area in the 129B6F1 males is statistically significant (p<0.05 by ANOVA).
(TIF)

Figure S3 **Simple interval mapping of the initial C57BL/6J×SJL/J F2 cross.**
(TIF)

Figure S4 **Simple interval mapping of different measures of angiogenic response in the full C57BL/6J×SJL/J F2 cross.** The use of different measures of angiogenic response as the trait to be mapped was explored. AVA is the (unadjusted) average of vessel areas of the left and right eyes. AVL is the average of the vessel length of the left and right eyes. NORMAVA is the average vessel area divided by (normalized) the vessel area measured in C57BL/6J controls assayed at the same time.
(TIF)

Figure S5 **Effect of SJL alleles on vessel area in B6.SJL Chromosome 5 N6F1 animals.** Note the truncated vertical axis. None of these differences are statistically significant (ANOVA).
(TIF)

Figure S6 Effect of SJL alleles on vessel area in B6.SJL Chromosome 7 N8F1 animals. Note the truncated vertical axis.
(TIF)

Table S1 **Markers showing near-significant and significant association with bFGF-induced corneal neovascularization in the initial C57BL/6J×SJL/J F2 cross.**
(DOCX)
