# Supplementary figures and images for: The Classical Pink-Eyed Dilution Mutation Affects Angiogenic Responsiveness
Source: PLoS One. 2012 May 15;7(5):e35237. doi: 10.1371/journal.pone.0035237 (PMC3352893; doi:10.1371/journal.pone.0035237)

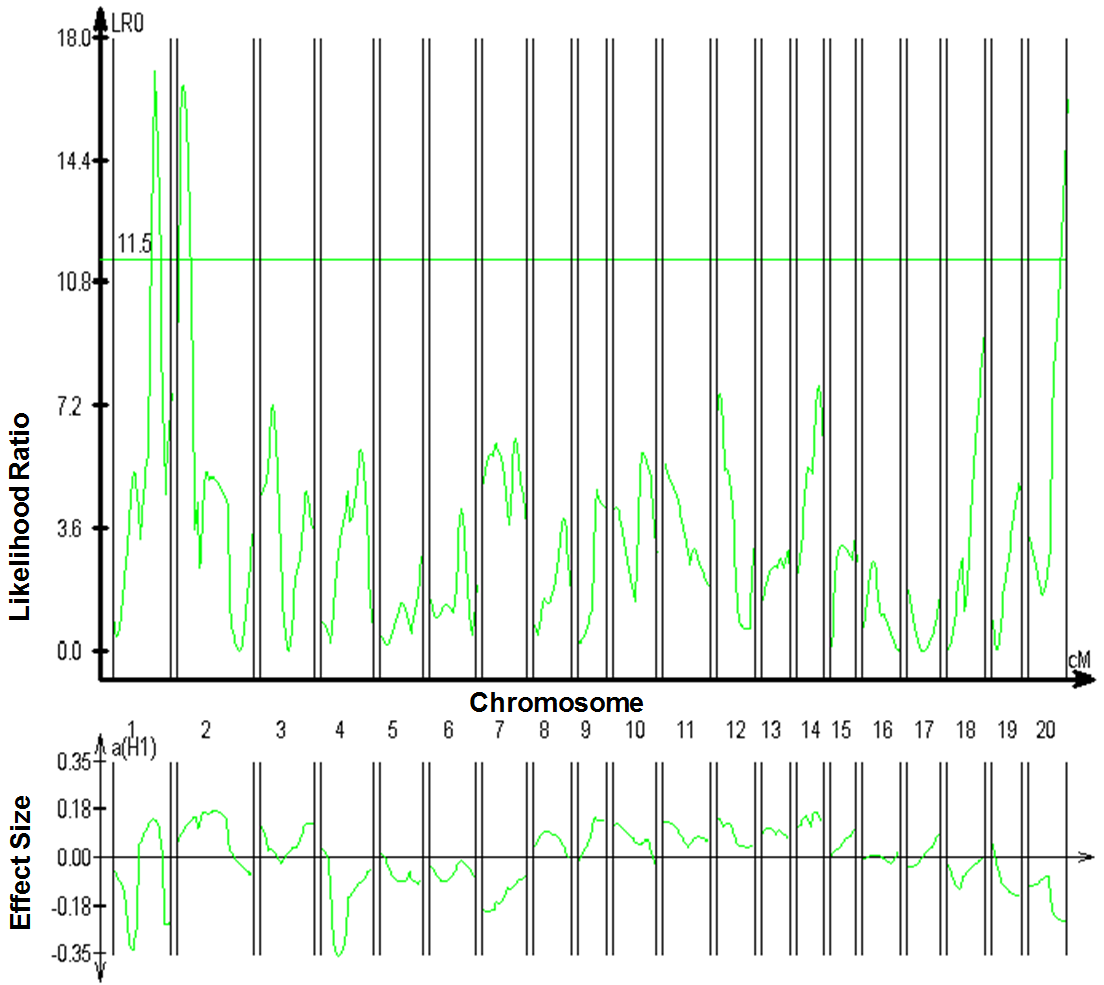

Supplement: Figure S1 — Composite interval mapping of the C57BL/6J×129P1/ReJ F2 cross. Whole genome composite interval map. Top: likelihood ratio statistic or likelihood of a region being linked to bFGF-induced corneal neovascularization. Bottom: predicted additive effect of a region on bFGF-induced corneal neovascularization. The indicated likelihood ratio of 11.5 is equivalent to a LOD score of 2.5. (TIF) [file pone.0035237.s001.tif]

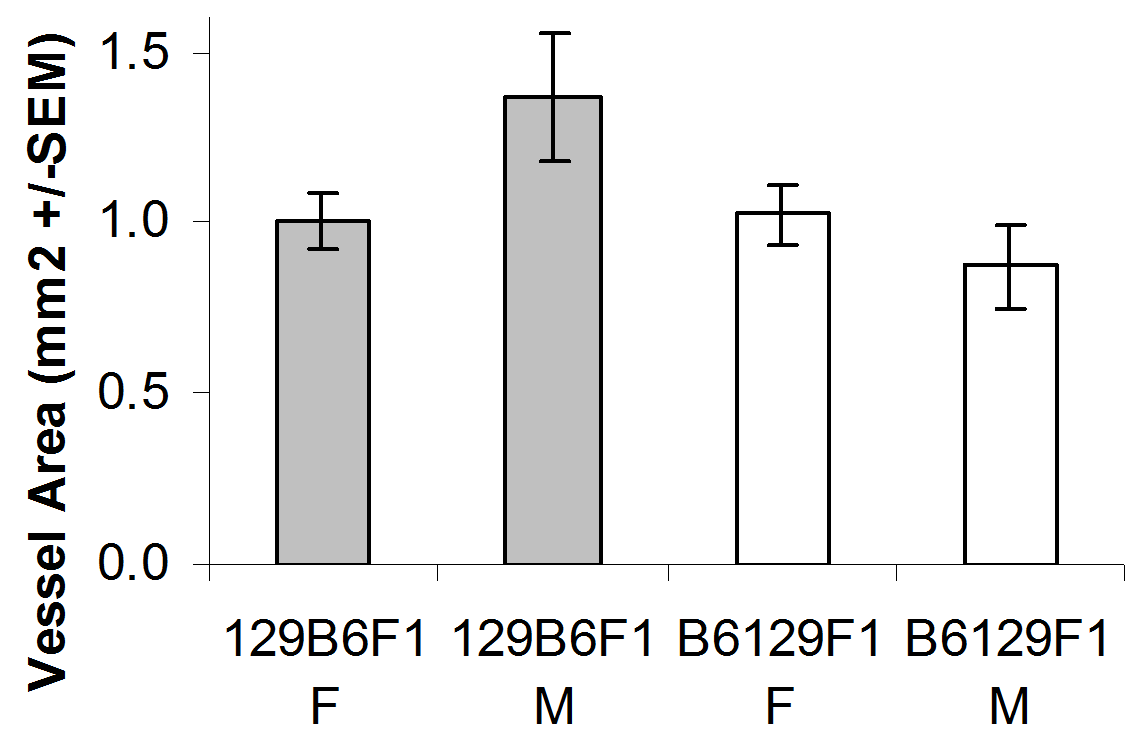

Supplement: Figure S2 — Effect of parental gender on vessel area in a 129×B6 F1 cross. Pellets contain 10 ng bFGF. The increase in vessel area in the 129B6F1 males is statistically significant (p<0.05 by ANOVA). (TIF) [file pone.0035237.s002.tif]

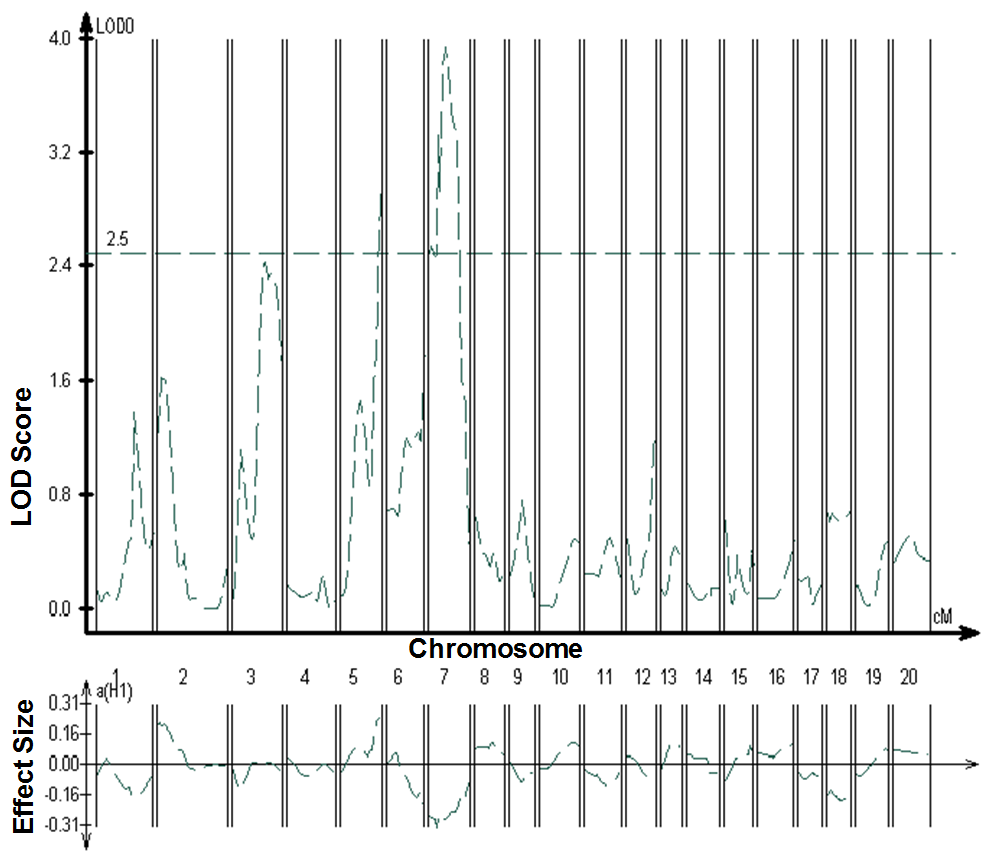

Supplement: Figure S3 — Simple interval mapping of the initial C57BL/6J×SJL/J F2 cross. (TIF) [file pone.0035237.s003.tif]

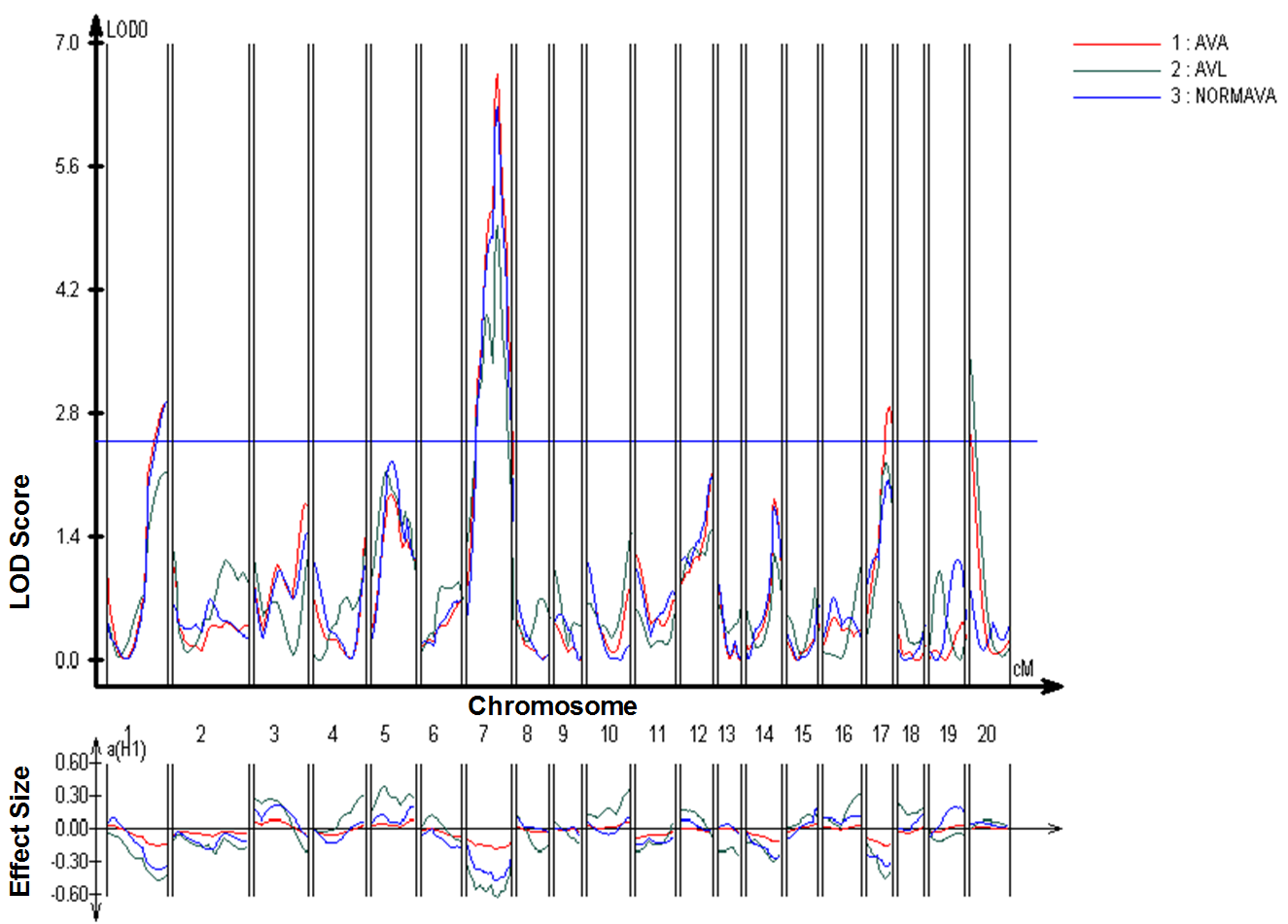

Supplement: Figure S4 — Simple interval mapping of different measures of angiogenic response in the full C57BL/6J×SJL/J F2 cross. The use of different measures of angiogenic response as the trait to be mapped was explored. AVA is the (unadjusted) average of vessel areas of the left and right eyes. AVL is the average of the vessel length of the left and right eyes. NORMAVA is the average vessel area divided by (normalized) the vessel area measured in C57BL/6J controls assayed at the same time. (TIF) [file pone.0035237.s004.tif]

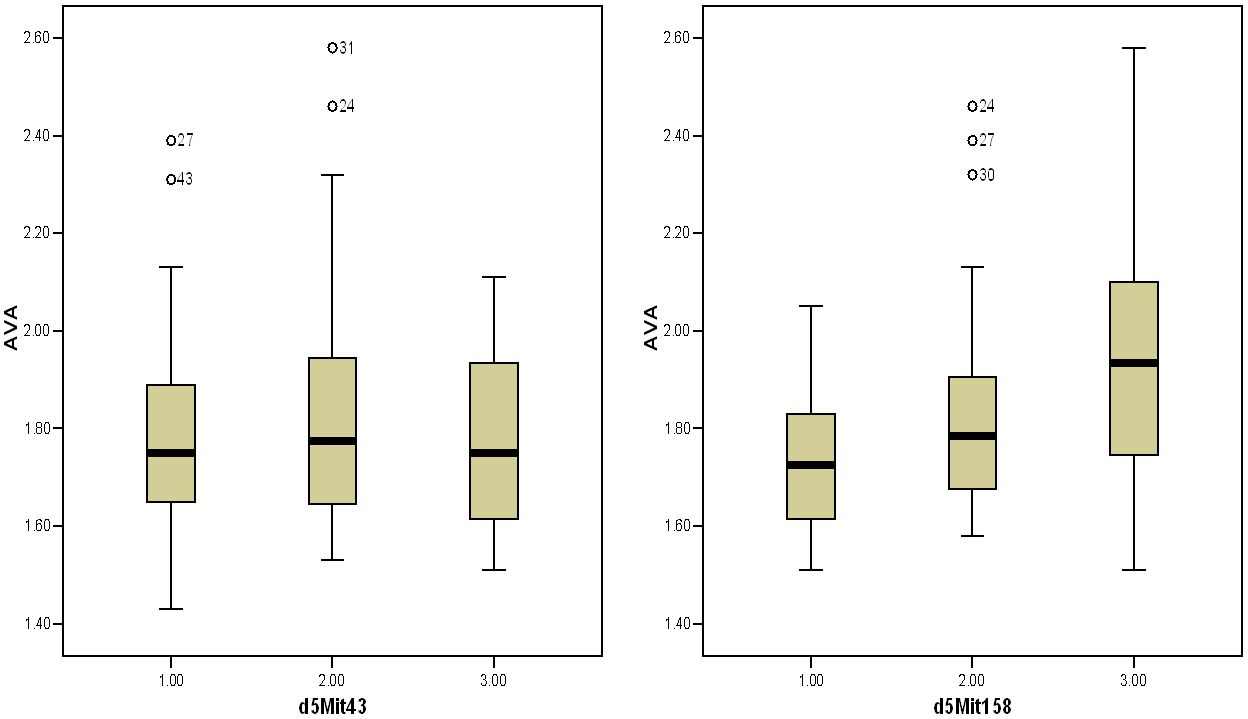

Supplement: Figure S5 — Effect of SJL alleles on vessel area in B6.SJL Chromosome 5 N6F1 animals. Note the truncated vertical axis. None of these differences are statistically significant (ANOVA). (TIF) [file pone.0035237.s005.tif]

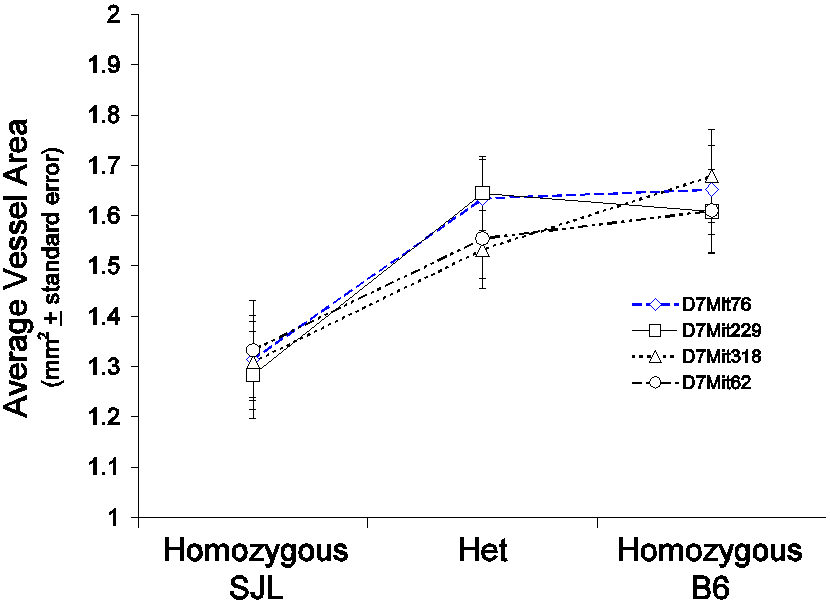

Supplement: Figure S6 — Effect of SJL alleles on vessel area in B6.SJL Chromosome 7 N8F1 animals. Note the truncated vertical axis. (TIF) [file pone.0035237.s006.tif]
