# Supplementary material for: The Classical Pink-Eyed Dilution Mutation Affects Angiogenic Responsiveness
Source: PLoS One. 2012 May 15;7(5):e35237. doi: 10.1371/journal.pone.0035237 (PMC3352893; doi:10.1371/journal.pone.0035237)
Supplement: Table S1 — Markers showing near-significant and significant association with bFGF-induced corneal neovascularization in the initial C57BL/6J×SJL/J F2 cross. (DOCX) [file pone.0035237.s007.docx]

| **Marker** | **Chrom.** | **cM** | **b0** | **b1** | **F(1,n-2)** | **pr(F)** |  |
| --- | --- | --- | --- | --- | --- | --- | --- |
| D2Mit464 | 2 | 9.5 | 1.67 | 0.108 | 3.229 | 0.073 |  |
| D3Mit106 | 3 | 55 | 1.67 | 0.128 | 4.215 | 0.041 | * |
| D5Mit205 | 5 | 45 | 1.668 | 0.128 | 5.229 | 0.023 | * |
| D5Mit338 | 5 | 59 | 1.668 | 0.089 | 2.495 | 0.115 |  |
| D5Mit158 | 5 | 62 | 1.667 | 0.104 | 3.351 | 0.068 |  |
| D5Mit188 | 5 | 64 | 1.667 | 0.124 | 5.214 | 0.023 | * |
| D5Mit168 | 5 | 78 | 1.666 | 0.179 | 11.4 | 0.001 | *** |
| D5Mit99 | 5 | 80 | 1.667 | 0.166 | 8.892 | 0.003 | ** |
| D6Mit149 | 6 | 46 | 1.67 | -0.098 | 3.04 | 0.082 |  |
| D6Mit59 | 6 | 67 | 1.667 | -0.126 | 5.265 | 0.022 | * |
| D6Mit294 | 6 | 73 | 1.666 | -0.147 | 7.049 | 0.008 | ** |
| D7Mit246 | 7 | 15 | 1.665 | -0.164 | 7.206 | 0.008 | ** |
| D7Mit270 | 7 | 18 | 1.663 | -0.191 | 11.42 | 0.001 | *** |
| D7Mit229 | 7 | 23 | 1.664 | -0.202 | 14.96 | 0 | *** |
| D7Mit145 | 7 | 26 | 1.664 | -0.209 | 15.87 | 0 | **** |
| Oca2 | 7 | 28 | 1.664 | -0.21 | 16.19 | 0 | **** |
| D7Mit318 | 7 | 37 | 1.664 | -0.194 | 14.91 | 0 | *** |
| ALBINO | 7 | 44 | 1.664 | -0.198 | 14.67 | 0 | *** |
| D7Mit126 | 7 | 50 | 1.665 | -0.141 | 7.234 | 0.008 | ** |
| D8Mit94 | 8 | 13 | 1.669 | 0.1 | 2.849 | 0.092 |  |
| D9Mit48 | 9 | 34 | 1.668 | -0.114 | 3.104 | 0.079 |  |
